# Formal Description of *Cladocopium proximale* sp. nov., a Coral Photosymbiont From the C1 Radiation

**DOI:** 10.1002/ece3.74569

**Published:** 2026-11-05

**Authors:** Giorgio Terzi, Madeleine J. H. van Oppen, Davide Seveso, Matthew R. Nitschke

**Affiliations:** ^1^ Department of Earth and Environmental Sciences (DISAT) University of Milano—Bicocca Milano Italy; ^2^ Australian Institute of Marine Science Townsville Queensland Australia; ^3^ School of BioSciences The University of Melbourne Parkville Victoria Australia; ^4^ MaRHE Center (Marine Research and High Education Centre) Magoodhoo Island Faafu Atoll Maldives; ^5^ School of Biological Sciences Victoria University of Wellington Wellington New Zealand

**Keywords:** ITS2, meta‐haplotype, phylogeny, psbAncr, symbiosis, taxonomy

## Abstract

Coral reefs are among the most biodiverse ecosystems on Earth and rely on endosymbiosis with Symbiodiniaceae, a diverse family of dinoflagellates essential for coral nutrition and which also play an important role in thermal tolerance. Despite their ecological importance, resolving species boundaries within Symbiodiniaceae remains challenging, partly due to limited phylogenetic signal in commonly used genetic markers and the absence of broadly accepted criteria for species delimitation. Refining taxonomic resolution is therefore critical for understanding symbiont diversity, distributions, and evolutionary relationships. In this study, we delimit two closely related *Cladocopium* species using a meta‐haplotype framework applied to two hypervariable loci—the ITS2 region and the *psbA* non‐coding region (psbA^ncr^). Slowly evolving markers failed to differentiate *Cladocopium proximale* (SCF049; described here) from its sister species *C. proliferum* . In contrast, consistent genetic differences between the two taxa were detected across both hypervariable regions. Together with morphological differences, these results support the recognition of *C. proximale* and *C. proliferum* as distinct species. Our results highlight the value of combining multiple hypervariable markers to improve taxonomic accuracy in Symbiodiniaceae, providing a stronger basis for understanding how symbiont diversity and ecological differentiation may influence coral resilience and responses to environmental change. More broadly, closely related Symbiodiniaceae such as these provide important systems for examining speciation processes, including potential ecological differentiation and reproductive compatibility.

## Graphical abstract

We formally describe *Cladocopium proximale* sp. nov., a coral symbiont indistinguishable from its sister species *C. proliferum* using conventional genetic markers. By integrating ITS2 and psbAncr meta‐haplotype analyses, we resolve one of the closest known species pairs in Symbiodiniaceae and reveal that this lineage has likely been previously misidentified. Our results provide a robust framework for delimiting cryptic symbiont diversity with direct implications for coral ecology and restoration.

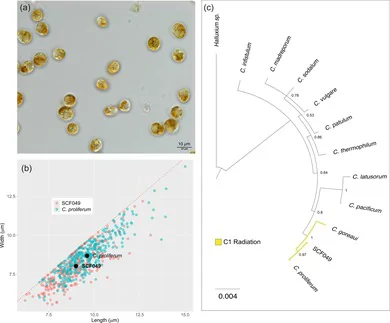

**Abbreviations**
AIMS: Australian Institute of Marine Science
ASV: amplicon sequence variant
Cob: cytochrome b
Cox1: cytochrome c oxidase subunit I
cp23S: chloroplast large ribosomal subunit (23S rRNA gene)
DIV: Defining Intragenomic Variant
GUniFrac: generalized UniFrac distance
HSD: honestly significant difference (Tukey's HSD test)
IDT: Integrated DNA Technologies
ITS2: Internal Transcribed Spacer 2
LSU: large ribosomal subunit
MCMC: Markov Chain Monte Carlo
MED: minimum entropy decomposition
NCBI: National Center for Biotechnology Information
NGS: next‐generation sequencing
PCoA: principal coordinate analysis
PCR: polymerase chain reaction
PERMANOVA: permutational multivariate analysis of variance
PERMDISP: permutational analysis of multivariate dispersions
psbAncr: *psbA* non‐coding region
SCF: Symbiont Culture Facility
SNP: single nucleotide polymorphism
UPGMA: unweighted pair group method with arithmetic mean
WEHI: Walter and Eliza Hall Institute

## 1 Introduction

Coral reefs are biodiversity hotspots providing millions of people with services such as food, livelihoods through fisheries and tourism, coastal protection, and holding cultural and spiritual values for some peoples (Hughes, Barnes, et al. 2017). These complex biogenic structures are primarily formed by scleractinian corals, which engage in a photosymbiotic relationship with unicellular dinoflagellate algae of the family Symbiodiniaceae (also referred to as zooxanthellae). Symbiodiniaceae translocate a significant portion of their photosynthates to the coral host, often fulfilling daily energetic requirements (Davies 1991; Muscatine et al. 1997). However, this symbiosis is highly sensitive to environmental stress. Anthropogenic climate change has led to rising sea temperatures which can trigger the breakdown of the coral‐algal relationship, a phenomenon known as coral bleaching (van Woesik et al. 2022; Wuitchik et al. 2024). If temperatures do not return to tolerable levels within a short period of time, corals face high mortality risks when in a bleached state. Since the first recorded global mass bleaching event in 1998 (Goreau et al. 2000), widespread coral loss has intensified (Hughes, Kerry, et al. 2017), making bleaching one of the most pressing threats to reef ecosystems in the Anthropocene.

A growing body of evidence highlights the key role that algal symbionts play during coral bleaching (Perez et al. 2001). Different symbiont species and strains respond differently to heat stress and shape the outcome of coral bleaching for the host (Berkelmans and van Oppen 2006). In fact, some genera of Symbiodiniaceae (e.g., *Durusdinium*) are generally recognized for their greater tolerance to heat stress and, in some cases, the symbiosis persists during heatwaves (Cunning and Baker 2020). Yet despite their ecological significance, our understanding of Symbiodiniaceae diversity and taxonomy remains incomplete. Symbiodiniaceae contain extensive cryptic diversity, and many species are difficult to distinguish due to subtle morphological differences and the reliance on multi‐copy molecular markers, factors that hinder clear species delineation (Kavousi et al. 2020). Moreover, many genetic analyses to date have been conducted on bulk community samples, which may contain multiple co‐occurring taxa, obscuring fine‐scale species boundaries and symbiotic specialization (Cunning et al. 2017).

While resolving Symbiodiniaceae taxonomy is fundamentally important for understanding natural diversity and coral‐symbiont interactions, it also carries increasing applied relevance, particularly as symbiont‐based interventions advance. In response to the decline of coral reefs, research effort is increasingly focused on strategies to slow or reverse this decline, including strategies that can be practically applied through ecological restoration (Buerger et al. 2020; Nitschke et al. 2024). One promising approach is assisted evolution, a range of interventions to accelerate adaptation to environmental change via naturally occurring evolutionary processes (van Oppen et al. 2015). Assisted evolution aims to enhance certain attributes such as temperature tolerance, growth and reproduction rates, among other traits. One approach within assisted evolution is Symbiodiniaceae manipulation, whereby heat‐sensitive symbiont strains or species are replaced with more heat‐tolerant ones to enhance coral thermal tolerance (Cunning and Baker 2020). This can involve the use of naturally heat‐tolerant Symbiodiniaceae as well as cultured symbionts whose thermal tolerance has been enhanced through experimental evolution. The latter approach has been successfully applied to several Symbiodiniaceae genera (Scharfenstein et al. 2024). Symbionts with enhanced thermal tolerance can subsequently be introduced into corals, with the aim of increasing their ability to persist under environmental stress (Nitschke et al. 2024). Accurate characterization of Symbiodiniaceae cultures used in such interventions is therefore important (Nitschke et al. 2024), particularly when they may ultimately be deployed onto reefs, where symbiont manipulation requires careful assessment of potential ecological trade‐offs and detrimental effects before implementation (National Academies of Sciences and Medicine 2019).

Species delimitation in Symbiodiniaceae is generally approached within a lineage‐based framework, in which species are regarded as independently evolving lineages supported by congruent evidence from multiple characters (Davies et al. 2023). Because reproductive isolation is difficult to assess directly in Symbiodiniaceae, the generally accepted minimum requirements for formal Symbiodiniaceae description are cell size and six phylogenetic markers, two for each nuclear (LSU, ITS2), mitochondrial (Cox1, Cob) and chloroplast (cp23s, psbA^ncr^) genomes (Davies et al. 2023). The combined use of slow‐ and fast‐evolving loci allows for a robust description of a species, both with respect to distant and closely related taxa (Lee et al. 2015) (Butler et al. 2023). Slow‐evolving markers, such as the large ribosomal subunit (LSU), the cytochrome c oxidase subunit I (Cox1), the cytochrome b, and the chloroplast large ribosomal subunit (cp23s), are regions of the genome under selection since they code for a functional product (Butler et al. 2023). Thus, these loci evolve slowly and variation among these markers typically reflect genus‐level divergence (LaJeunesse et al. 2018). Fast‐evolving markers, such as the multi‐copy and hypervariable Internal Transcribed Spacer 2 (ITS2) and the non‐coding region of the photosystem II reaction center protein D1 gene (psbA^ncr^), are neutral loci that harbor a constant rate of mutation (Smith et al. 2017) (LaJeunesse and Thornhill 2011). These regions generally exhibit genetic variation even among closely related species and allow resolution at the species and sub‐species level (LaJeunesse 2002).

The multi‐copy nature of the ITS2 and psbA^ncr^ loci is differently structured within a symbiont cell. ITS2 is a region of the rDNA cistron repeated in tandem in potentially hundreds of copies (Arif et al. 2014; Saad et al. 2020). The process of concerted evolution, where mechanisms like unequal crossing over and gene conversion ensure that all ITS2 copies within an individual species remain nearly identical, is relaxed in the ribosomal cistron of Symbiodiniaceae, meaning that ITS2 variants accumulate (Koch et al. 2003). This relaxation allows for the existence of intra‐genomic diversity, which can be misinterpreted as evidence of genetic exchange between populations or even hybridization (i.e., reproduction between genetically distinct lineages). However, in the former, the presence of multiple shared ITS2 variants among taxa does not necessarily indicate gene flow, but rather reflects shared ancestral polymorphism as a consequence of incomplete lineage sorting (Stat et al. 2011; Stern et al. 2012). As a result, while ITS2 remains valuable for species delimitation, its intra‐genomic variability necessitates cautious interpretation when inferring evolutionary relationships among Symbiodiniaceae populations (Wilkinson et al. 2015). In a similar way, psbA^ncr^ is found in the plastid minicircles, of which multiple copies exist within each symbiont cell and which can undergo recombination; this leads to the presence of multiple psbA^ncr^ variants within the same symbiont cell (Moore et al. 2003). The complex patterns of genetic variation in multi‐copy markers can be deciphered by using a multi‐gene high‐throughput sequencing approach which detects all the variants to generate a “meta‐haplotype” for each psbA^ncr^ and ITS2 (Denis et al. 2024; Smith et al. 2017).

*Cladocopium* (formerly *Symbiodinium* Clade C) is the most abundant and diverse genus of Symbiodiniaceae LaJeunesse (2004). In this study, we consider the case of two closely related *Cladocopium* species, one already formally described (*Cladocopium proliferum*; (Butler et al. 2023)) and the other hypothesized to be a new species. We formally describe the new species based on cell morphology and genetic divergence from *C. proliferum* . Based on our findings, we propose a standardized approach for differentiating closely related Symbiodiniaceae taxa, which complements the established ITS2 haplotyping method by incorporating an additional psbA^ncr^ metabarcoding step.

## 2 Material and Methods

### 2.1 Culture Material, Coral Voucher Material, and Published Data

The two cultures compared here, SCF049.01 (hereafter referred to as SCF049) and SCF055.01 (the type strain of *C. proliferum* , hereafter referred to as SCF055), are maintained in the Symbiont Culture Facility (SCF) at the Australian Institute of Marine Science (AIMS), and reference sequences used to delimit them were obtained from these cultures. SCF055 is assumed to be an isoclonal culture isolated from an *Acropora kenti* (formerly *Acropora tenuis* ) colony at Magnetic Island, Great Barrier Reef. SCF049 is assumed to be an isoclonal culture isolated from a *Pocillopora damicornis* colony from Davies Reef. Additional *C. proliferum* sequences for four conserved markers (see below) were retrieved from previously published data on the wild sample A03_50_C1 (Butler et al. 2023), also collected from an *A. kenti* colony in Rib North Reef on the Great Barrier Reef. Moreover, to broaden the pool of samples used to test our ITS2/psbA^ncr^ meta‐haplotype approach we obtained ITS2 sequences from AIMS voucher samples Gal‐509 (*Galaxea* sp.), and Psa‐36, Psa‐44, Psa‐109 and Psa‐113 (*Psammocora cf columna*). Metadata for *Galaxea* and *Psammocora* samples are shown in Table S3.

### 2.2 Cell Culture

Cultures SCF049 and SCF055 were grown in IMK medium and maintained at a constant temperature of 27°C under approximately 50 μmol photons m^2^ s^−1^ of photosynthetically available radiation on a 12:12 h light: dark cycle.

### 2.3 Size Measurement

From each culture, 300 cells were imaged using bright‐field microscopy with a Zeiss inverted microscope at 96× magnification. To ensure consistency, the images of the symbiont cells were taken at the same time of the day for both cultures and at the same number of days following sub‐culturing. Width and length measurements were taken using ImageJ software, relating pixels to known distances. In R, mean width and length values were calculated with 95% confidence intervals. A *t*‐test at 95% confidence was used to statistically test for a difference in width and length between the two cell populations.

### 2.4 DNA Extraction, Amplification and Sequencing

Wayne's protocol was used to extract genomic DNA from 1 mL samples collected from each culture (approximately 1 × 10^6^ cells) in three technical replicates (Damjanovic et al. 2019). The same protocol was applied to coral voucher specimens with approximately 2–3 mm^2^ of tissue. DNA extracts were run on a 1% agarose/1× TAE gel to check for integrity. Primers used for PCR amplification of the six markers LSU, COX1, COB, cp23S, psbA^ncr^, and ITS2 are listed in Table 1. All amplicons were visualized on a 1% agarose/0.5× TBE gel to check amplification. After purification through Sephadex G‐50 resin columns, amplicons of five markers (LSU, COB, COX1, cp23S, psbA^ncr^) were Sanger sequenced (Macrogen, Korea), and the ITS2, psbA^ncr^ amplicons were prepared for next‐generation sequencing (NGS).

**TABLE 1:** Primer sequences for amplification of all the DNA regions investigated in this study.

Primer	Sequence	References	Expected size (bp)
LSU‐Fwd	CCCGCTGAATTTAAGCATATAAGTAAGCGG	Zardoya et al. ( 1995)	~600
LSU‐Rev	GTTAGACTCCTTGGTCCGTGTTTCAAGA	Zardoya et al. ( 1995)	
Cox1‐Fwd	AAAAATTGTAATCATAAACGCTTAGG	Zhang et al. (2008)	~950
Cox1‐Rev	TGTTGAGCCACCTATAGTA AACATTA	Zhang et al. ( 2008)	
Cob‐Fwd	ATGAAATCTCATTTACAWWCATATCCTTGTCC	Zhang et al. ( 2008)	~900
Cob‐Rev	TCTCTTGAGGKAATTGWKMACCTATCCA	Zhang et al. ( 2008)	
cp23s‐Fwd	GGCTGTAACTATAACGGTCC	Zhang et al. ( 2000)	~550
cp23s‐Rev	CCATCGTATTGAACCCAGC	Zhang et al. ( 2000)	
psbAncr‐Fwd	GCATGAAAGAAATGCACACAACTTCCC	Moore et al. ( 2003)	~1500
psbAncr‐Rev	GGTTCTCTTATTCCATCAATATCTACTG	Moore et al. ( 2003)	
ITS2‐Fwd + MiSeq Adapter	TCGTCGGCAGCGTCAGATGTGTATAAGAGACAGGAATTGCAGAACTCCGTGAACC	Hume et al. ( 2018)	~300
ITS2‐Rev + MiSeq Adapter	GTCTCGTGGGCTCGGAGATGTGTATAAGAGACAGCGGGTTCWCTTGTYTGACTTCATGC	Hume et al. ( 2018)	
psbAncr_NGS_Fwd + MiSeq Adapter	GTGACCTATGAACTCAGGAGTCCCGAGCAAAATAACCAAGC	This study (adapted from Hume et al. 2015)	~300
psbAncr_NGS_Rev + MiSeq Adapter	CTGAGACTTGCACATCGCAGCGTTGTGGATAGCACGAAT	This study (adapted from Hume et al. 2013 )	

*Note:* Underlined regions indicate adaptor sequences for metabarcoding.

ITS2 amplicons were library‐prepped for sequencing at The Walter and Eliza Hall Institute WEHI Illumina MiSeq platform following the protocol by (Dungan et al. 2021). Following multiple sequence alignment of the psbA^ncr^ from preliminary Sanger data, custom psbA^ncr^ primers were developed targeting two conserved regions identified across SCF049, SCF055, and other C1 radiation sequences. The primers were designed to produce an amplicon size of approximately 300 bp suited to Illumina NGS. The reaction conditions were 95°C for 15 min; then 30 cycles of 94°C for 30 s, 60°C for 90 s and 72°C for 30 s; followed by a single extension at 72°C for 10 min. The psbA^ncr^ amplicons were library prepared and sequenced as above.

### 2.5 Sequence Data Analysis

#### 2.5.1 Sanger Sequence Data

All markers except for psbA^ncr^ yielded a high‐quality sequence for both forward and reverse Sanger sequencing reactions. The following sequence quality control and alignment steps were conducted in R using the R packages *DECIPHER* (Wright 2016), *bioseq* (Keck and Mahon 2020), *Biostrings* (Pages et al. 2013), and *ape* (Paradis and Schliep 2019).

For the four slow‐evolving markers, Sanger chromatograms were quality checked, reverse sequences were reverse complemented, aligned with the forward sequence, and a consensus sequence allowing for mismatches (utilizing IUPAC degenerate bases) was generated. These were then further aligned with other *Cladocopium* species sequences from (Butler et al. 2023), accessed from the NCBI GenBank nucleotide database. Sequences from the genus *Halluxium*, which is the sister lineage to *Cladocopium*, were used as an outgroup to root the tree (Nitschke et al. 2020). GenBank accession numbers for all previously published sequences utilized here are reported in Table S1. Poorly aligned and protruding regions at the extremities of the sequences were trimmed. Sequences of the four loci from the same species were concatenated and a Bayesian Inference tree was generated using MrBayes v.3.2.1, implementing the General Time Reversible model where indels were treated as missing data. Each Markov Chain Monte Carlo (MCMC) analysis was run for 10^6^ generations total and sampled every 100 generations. The first quarter of trees was discarded as burn‐in corresponding with the convergence of chains (Butler et al. 2023). Branch support was estimated using Bayesian posterior probability.

For psbA^ncr^ Sanger sequencing data, the expected 1200 bp amplicon returned only a ~250 bp region at the 3′ of sufficient quality for phylogenetic analysis. Once this region was aligned with sequences from other *Cladocopium* species including *C. proliferum* , *C. latusorum*, *C. pacificum* , and *C. vulgare* (Butler et al. 2023), it was used for more detailed inter‐ and intra‐species analysis. GenBank accession numbers for all previously published psbA^ncr^ sequences utilized here are reported in Table S2. In addition, we included the five most abundant amplicon sequence variants (ASVs) from our Illumina dataset (see below), each covering the same region, as well as two new consensus sequences for SCF049 and *C. proliferum* SCF055, generated by Sanger sequencing amplicons produced with internal primers (Forward primer as the forward for the Illumina library and reverse primer as the reverse for the psbA^ncr^ full‐length amplification); we refer to this region hereafter as “Sanger internal.” *C. goreaui* was excluded due to the absence of this 250 bp region in public databases (psbA^ncr^ full length alignment shown in Figure S1). A Bayesian Inference tree was generated using MrBayes v.3.2.1, implementing General Time Reversible model as above.

#### 2.5.2 Illumina MiSeq Data

ITS2 demultiplexed forward and reverse Fastq files were submitted to SymPortal.org for analysis of “Defining Intragenomic Variants” (DIVs) and ITS2 type profiles (Hume et al. 2019). psbA^ncr^ MiSeq data were subject to demultiplexing, adapter and primer removal using *cutadapt*, followed by quality filtering, denoising, and chimera removal in R using the DADA2 package to produce amplicon sequence variants or “ASVs” using default settings. The resulting psbA^ncr^ ASVs were aligned and a feature table for each sample with ASVs and their relative abundance was constructed. A k‐mer approach was adopted to produce a pairwise sequence comparison from which UPGMA trees for both ITS2 and psbA^ncr^ hypervariable regions were generated according to k‐mer = 5 + GUniFraq (0.5) distances. From this, multi‐gene congruent “meta‐haplotypes” among samples were inferred (Marzonie et al. 2024).

### 2.6 Secondary Structure

To investigate potential artifact formation during PCR amplification, we predicted the secondary structures of the SCF049 psbA^ncr^ ASV1 sequence and compared this to the full‐length SCF055 psbA^ncr^ sequence. Consensus FASTA sequences were used as input for RNAstructure online software (version 6.0.1), using default parameters and the DNA folding option. For the SCF055 full‐length psbA^ncr^ sequence, a particularly complex hairpin‐like region identified in the predicted secondary structure was extracted and further analyzed using the IDT OligoAnalyzer Tool (Integrated DNA Technologies Inc.) to calculate its melting temperature (*T*_m_).

### 2.7 Data Mining for SCF055 and SCF049 ITS2 Profiles Among Published Symportal Studies

We used postMED (Minimum Entropy Decomposition) sequences from SymPortal to search for SCF049‐ and SCF055‐like ITS2 profiles from previously published datasets. SymPortal integrates quality‐controlled sequences into a centralized database and applies a standardized nomenclature system. Sequences are parsed into Defining Intragenomic Variants (DIVs), from which ITS2 type profiles are generated as discrete biological entities. To characterize the ecological and taxonomic context of putative symbiont lineages, we queried the SymPortal database for ITS2 type profiles matching the characteristic DIV composition and relative abundances of our samples.

The filtering parameters were (i) presence of the first 9 most abundant DIVs shared in both profiles (“C1,” “C1b,” “C1c,” “C42.2,” “C1bh,” “C1br,” “C72k,” “C1w,” “C1al”), and presence of C1 as the most abundant sequence in the profile. Once profiles with these characteristics were isolated, we opted for a k‐mer based approach to produce pairwise sequence comparisons of the filtered SymPortal ITS2 sequences (Fujise et al. 2021). We then produced a UPGMA dendrogram of ITS2 sequences. This tree was then input into a Generalized UniFrac function along with sequence abundances (GuniFrac, α = 0.5), a method considered to apply fair weighting to both rare and abundant sequences (Chen et al. 2012; Marzonie et al. 2024) when generating inter‐sample distances. GuniFrac distances were also used to generate a PCoA and visualize genetic distances between samples in a two‐dimensional space. Moreover, for each sample, we calculated the ratio of genetic distance from SCF049 and SCF055 ITS2 reference sequences (using the GuniFrac distance matrix). For visualization purposes, samples were grouped according to their relative distance from the SCF049 and *C. proliferum* reference profiles. Samples with distance ratios of 0–0.45 and 0.55–1 were descriptively labeled as SCF049‐like and *C. proliferum* ‐like, respectively, while samples with ratios of 0.45–0.55 were considered intermediate, reflecting their approximately equidistant position relative to the two reference profiles. The 0.45 and 0.55 thresholds defining the width of the intermediate range were selected heuristically to facilitate the visualization and description of patterns in the dataset and do not represent biologically or statistically defined species boundaries. Accordingly, these categories were not used for formal species assignment, but rather to explore whether ITS2 profiles resembling the two reference cultures occur among previously published wild samples.

### 2.8 Test of Symbiosis Establishment With *Acropora*

As a complementary qualitative assessment of symbiosis establishment, we tested whether the two closely related species were both capable of establishing an initial symbiosis with the same coral host. This experiment was intended to provide preliminary biological context alongside their taxonomic characterization, rather than a quantitative assessment of host compatibility. *Acropora kenti* coral colonies spawned on the 27th July 2024 during out‐of‐season spawning at the Sea Simulator facility at the Australian Institute of Marine Science (Koukoumaftsis et al. 2025). Two days postfertilization, viable larvae were moved to 6‐well plates containing 10 mL of filtered seawater and either SCF049 or SCF055 symbionts at a 10,000 cells mL^−1^ density in three technical 6‐well plate replicates for each symbiont. Larvae were at a 1 larvae mL^−1^ density and were placed at 27°C under approximately 50 μmol photons m^2^ s^−1^ of photosynthetically available radiation on a 12:12 h light: dark cycle. Seawater was sourced from Cape Cleveland, Queensland, Australia, and filtered to 1 μm prior to use in the experiment. Two larvae/well were examined and photographed for the presence of symbionts within their tissues at 24, 48, 72 h, and 6 days post‐exposure using a Zeiss inverted microscope under UV light. All examined larvae were imaged (144 larvae/symbiont treatment, 288 in total).

The SymPortal data‐mining analysis and the experimental assessment of symbiosis establishment were used to provide complementary ecological and biological context for the two closely related lineages. Ecological differentiation, such as differences in host associations or geographical distributions, can provide additional evidence supporting species boundaries; however, distinct Symbiodiniaceae species may also exhibit broad and overlapping ecological associations, particularly in host‐generalist lineages (Butler et al. 2023; Davies et al. 2023). Consequently, ecological differentiation cannot be considered a necessary or diagnostic criterion for distinguishing independently evolving Symbiodiniaceae lineages.

## 3 Results

### 3.1 Morphology

Length and width of 300 cells for both SCF049 and SCF055 (hereafter *C. proliferum* ) were measured (Figure 1). Although overlapping in size, there was statistically significant variation between the two cell populations, both in length and width (*p* < 0.001). SCF049 and *C. proliferum* length and width mean values were: 8.99 μm (±0.13 μm; CI), 8.03 μm (±0.13 μm; CI) and 9.61 μm (±0.14 μm; CI), 8.69 μm (±0.13 μm; CI), respectively. Thus, SCF049 is slightly smaller than SCF055 in both length and width by less than one micrometer.

**FIGURE 1:**
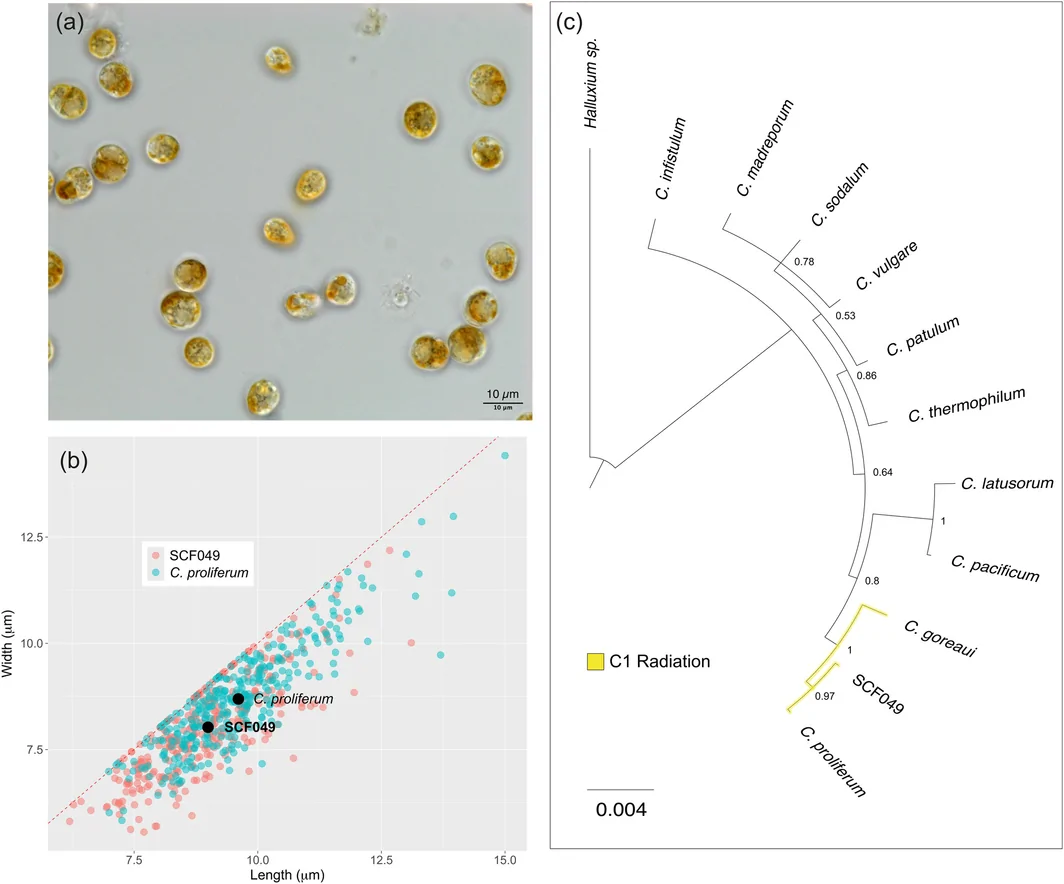
Morphological and phylogenetic characterization (LSU, COB, COX1, cp23S) of SCF049 in comparison with *C. proliferum* and other *Cladocopium* members. (a) Representative light microscopy image of SCF049 cells. (b) Scatterplot of width and length values for SCF049 and *C. proliferum* cells. The dashed line indicates 1:1 width‐length ratio. Black dots represent mean values for both species. (c) Bayesian inferred phylogenetic tree of the four slow‐evolving markers LSU, Cob, Cox1 and cp23S for SCF049.01, *C. proliferum* , and nine members of the *Cladocopium* genus. *Halluxium* represents an outgroup to all other Symbiodiniaceae genera. Branch support is estimated using Bayesian posterior probability.

### 3.2 Phylogenetics

#### 3.2.1 Slow Evolving Markers

The “slow evolving” markers, LSU, Cox1, Cob, and cp23S, showed no genetic difference between SCF049 and *C. proliferum* , confirming their close relatedness. As expected, the two species are also closely related to *Cladocopium goreaui* within the C1 radiation, which represents the sister species of the SCF049 and *C. proliferum* cluster. Contrary to previous reports, in our analysis *C. vulgare* did not cluster within the clade comprising *C. goreaui* , *C. proliferum* , and SCF049 (Butler et al. 2023).

#### 3.2.2 Rapidly Evolving Markers

To further investigate the genetic difference between SCF049 and *C. proliferum* , a meta‐haplotype approach of the ITS2 region was used. The ITS2 profile of SCF049 was found to contain multiple variants identical to those of *C. proliferum* (Figure 2). However, we discovered five DIVs in the SCF049 profile that were not present in *C. proliferum* , and the *C. proliferum* profile comprised four DIVs that were absent from the SCF049 profile. All these diagnostic sequences occurred in low relative abundance (Figure 2) except for C1cb, which was the seventh most abundant DIV in the *C. proliferum* profile and was absent in SCF049 (Figure 2). C1 was the most abundant DIV in both profiles, but for SCF049 its relative abundance was higher (around 70%). In addition, the second and third most abundant DIVs were inverted in order among the two symbionts, being C1c/C1b for SCF049 and C1b/C1c for *C. proliferum* . Other numerically abundant DIVs, such as C42.2, C1bh, and C72k, occurred in different proportions in the two haplotypes. These results are not attributable to sequencing artifacts, and they have been independently verified by a different laboratory group that maintains copies of the SCF049 culture (Maire et al. 2024).

**FIGURE 2:**
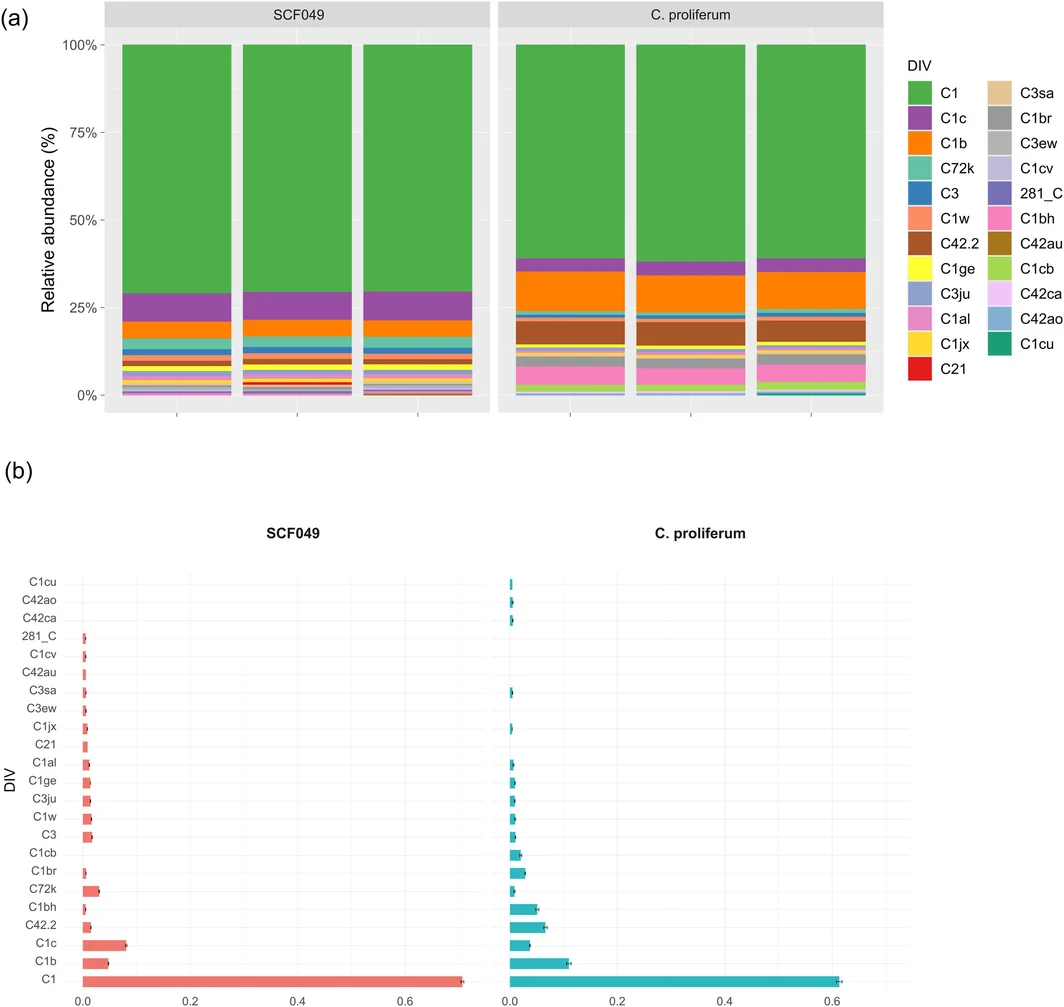
ITS2 type profiles of SCF049 and *C. proliferum* . (a) ITS2 type profile for SCF049 and *C. proliferum* , with bars representing the results of three technical replicates from the same culture. (b) comparison of presence and relative abundance of each DIV composing the two profiles. Error bars represent the standard deviation of the technical replicates.

Because Sanger sequencing of the psbA non‐coding region did not yield reliable results for the full length of the amplicon (see next section), we applied a targeted metabarcoding approach to characterize psbA^ncr^ diversity in SCF049 and *C. proliferum* . The only difference between the two species was observed in the relative abundance of ASVs shared in the two profiles, rather than in the presence of unique variants (Figure 3). ASV1 and ASV2, although present in low abundance in *C. proliferum* , accounted for around 80% of the total reads detected in SCF049. Conversely, ASV3 and ASV4, present at only background levels (less than 5%) in SCF049, were highly abundant in *C. proliferum* , accounting for 80% of the profile.

**FIGURE 3:**
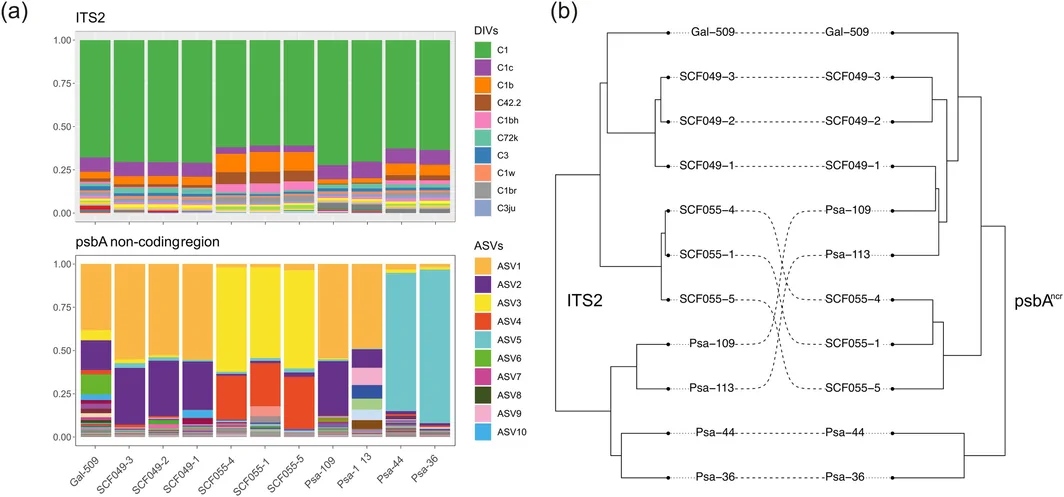
ITS2 and psbA^ncr^ haplotypes and comparative phylogeny. (a) ITS2 and psbA^ncr^ metabarcoding data. DIV profiles for the two markers of the same samples (aligned vertically across facets) were compared for three technical replicates of the two cultures SCF049 and SCF055 ( *C. proliferum* ) and five wild coral samples. The order of the samples is informed by the tanglegram in (b). (b) Tanglegram of UPGMA trees (k‐mer = 5 + GUniFraq (0.5) distances) of both ITS2 and psbA^ncr^ haplotypes for three technical replicates of the two cultures SCF049 and SCF055 and five wild coral samples.

We then tested the ability of the ITS2‐psbA^ncr^ meta‐haplotype approach to resolve phylogenetic assignments by applying it to symbiont communities in five wild coral samples belonging to the genera *Galaxea* (Gal‐509) or *Psammocora* (Psa‐109, Psa‐113, Psa‐36 and Psa‐44). The ITS2 profile was characteristic of SCF049, except for the samples Psa‐36 and Psa‐44 which presented as intermediate of SCF049 and *C. proliferum* (Figure 3). The psbA^ncr^ data confirmed the SCF049‐like nature of the *Galaxea* sample and two *Psammocora* samples (Psa‐109 and Psa‐113), but for the other *Psammocora* samples (Psa‐36 and Psa‐44) we found a unique intra‐genomic variant (ASV5) in very high abundance that was distinct from SCF049 and *C. proliferum* psbA^ncr^ haplotypes (Figure 3).

A test of multi‐gene congruence using a tanglegram of ITS2 and psbA^ncr^ UPGMA trees confirmed the distinct nature of SCF049 and *C. proliferum* , and that Psa‐36 and Psa‐44 potentially harbor a third distinct taxon. It also confirmed that Gal‐509 is similar to SCF049. The psbA^ncr^ haplotype of Psa‐109 and Psa‐113 was genetically similar to SCF049. These patterns could not be resolved through ITS2, which placed these samples as outgroups to both SCF049 and *C. proliferum* .

To assess congruence between sequencing approaches and verify the psbA^ncr^ signal detected by Illumina data, we next analyzed previously generated Sanger sequences for this region in SCF049 and *C. proliferum* . Due to template variability in our Sanger reaction leading to low‐quality chromatograms (likely indicating inter‐minicircle variability), only a ~250 bp segment near the 3′ end yielded clean and usable reads, but only for the reverse strand. While the resulting phylogeny largely showed species‐specific clustering, one notable inconsistency emerged: the Sanger sequence of SCF049 and *C. proliferum* generated with the flanking primers (i.e., the usable 250 bp at the 3′‐end of the full‐length amplicon) clustered outside of the group formed by both the Illumina ASVs and the Sanger sequence generated with internal primers (Sanger Internal, Figure 4), which greatly overlap. Sequence alignment revealed that this discrepancy coincided with a poorly aligned, palindromic, C‐rich region inside the 250 bp fragment of the psbA^ncr^, where previously published sequences also showed gaps (Figure 4). To investigate whether the 3′‐end 250 bp of the full‐length sequences were artifactual, we conducted a BLAST search against our high‐depth (500,000 to 2,000,000 reads per sample) Illumina sequences, which also include C‐rich region of the psbA^ncr^. No exact matches were found. In contrast, the Sanger Internal sequences generated with internal primers were a complete match to the ASV1/ASV2 and ASV3/ASV4 obtained with Illumina sequencing for SCF049 and *C. proliferum* , respectively. To explore the validity of the Sanger sequences further, we analyzed previously published full‐length psbA^ncr^ sequences generated through PCR and Sanger sequencing using RNAstructure software (Figures S2 and S3). Consistently, the C‐rich region displayed a high degree of structural complexity, overlapping exactly with the locations of indels in the full‐length amplicons. Using IDT's OligoAnalyzer tool, the thermodynamic potential of the harpin‐like secondary structure associated with the C‐rich region exhibited a melting temperature (*T*_m_) of 59.9°C, higher than the annealing temperature (55°C) used in the flanking primers protocol reported by Moore et al. (2003). In contrast, the shorter ~250 bp internal amplicon (Sanger Internal) had a comparatively simple secondary structure, with the full‐length non‐coding region (sample proliferum‐055) having a Δ*G* = −264 kcal/mol, whereas the internal amplicon (computed for ASV1) had a Δ*G* = −33.8 kcal/mol. These thermodynamic differences suggest that the structurally complex full‐length sequence is more prone to PCR artifacts, and that the simpler internal region is therefore the more reliable representation of the true sequence.

**FIGURE 4:**
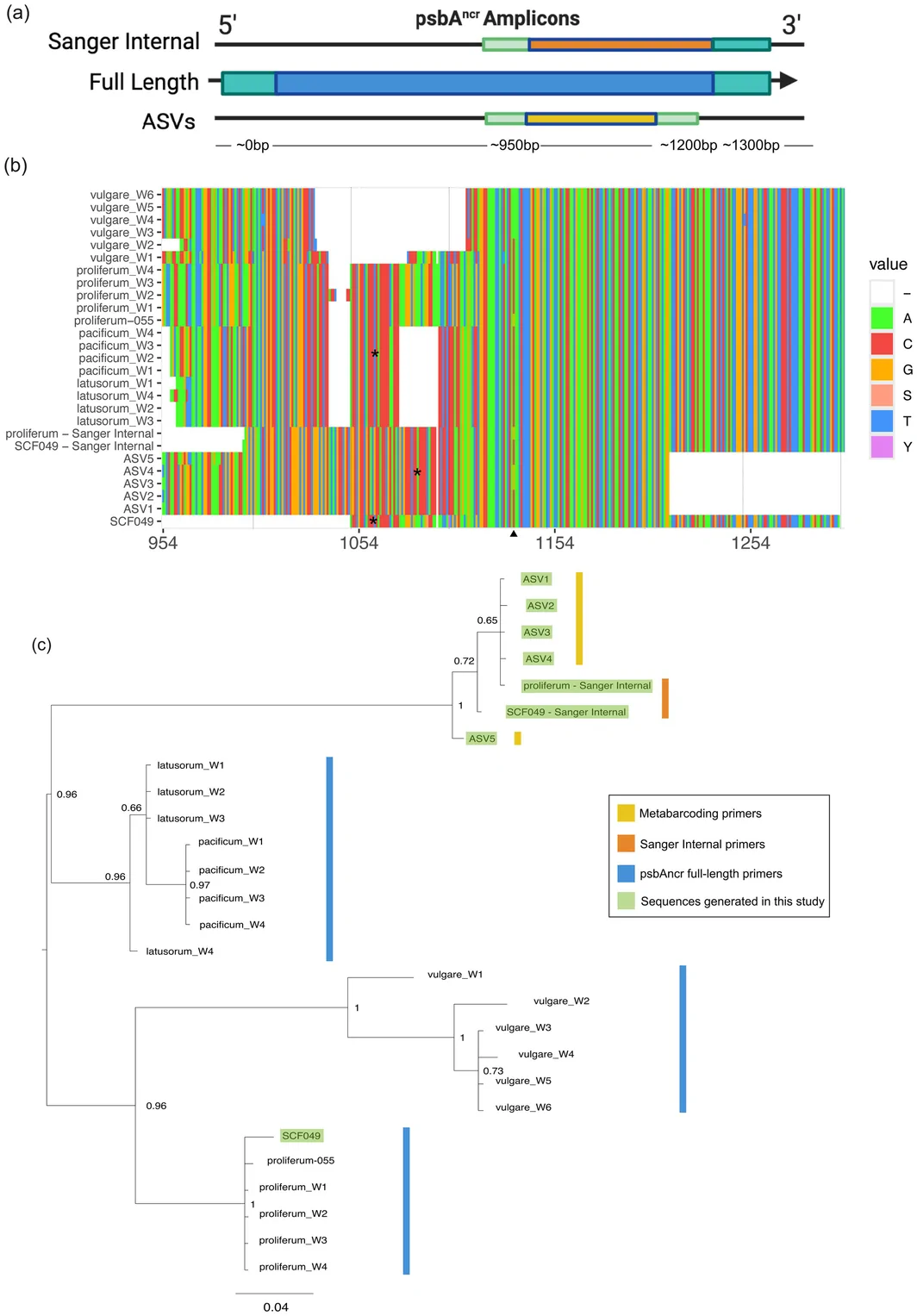
Sanger and Illumina sequencing results of the psbA^ncr^ region. (a) Primer design and respective amplification products within the psbA non‐coding region. Colors are used to differentiate primers and amplicons (Table 1). (b) Multiple sequence alignment of a 350 bp internal region used to generate the psbA^ncr^ Bayesian inferred phylogenetic tree. The black arrow indicates the diagnostic SNP between SCF049 and *C. proliferum* . Black asterisks indicate the C‐rich palindromic motif likely causing polymerase slippage and artefactual PCR products. (c) Bayesian inferred phylogenetic tree of the ~350 bp targeted region of the psbA^ncr^. Branch support is estimated using Bayesian posterior probability. Colors bars flanking each sequence refer to the amplicon generated (with respect to panel a) and the consequent primer set used.

Finally, we identified a single, highly consistent polymorphic site distinguishing SCF049 from *C. proliferum* : a cytosine (C) variant dominant in SCF049 and an adenine (A) variant dominant in *C. proliferum* . This diagnostic SNP was consistently detected across replicates and sequencing methods. In the psbA^ncr^ phylogeny, the sequence retrieved from the reference strain of *C. proliferum* clustered with all previously described environmental samples deposited as *C. proliferum* in Butler et al. (2023) (Figure 4). SCF049 grouped as a sister taxon within the same cluster. Notably, however, all environmental samples previously described as *C. proliferum* in this cluster carried the SCF049 allele at the diagnostic site, rather than the *C. proliferum* variant (Figure 4).

### 3.3 SymPortal Screening—Host Range

A SCF049 and *C. proliferum* ITS2 profile search against previously published studies that utilized the SymPortal analytical pipeline retrieved 37 wild coral symbiont samples from six studies (Camp et al. 2020; Ziegler et al. 2017; Camp et al. 2019; Lewis et al. 2022; Leinbach et al. 2023). Using the descriptive categories defined above, samples showing greater similarity to the *C. proliferum* reference profile were associated only with *Acropora*, whereas samples showing greater similarity to the SCF049 reference profile occurred in *Acropora*, *Galaxea*, *Astreopora*, and *Psammocora*, as well as the hydrozoan *Millepora*. These associations should be interpreted as preliminary ecological patterns rather than species‐level assignments, as the categories are based solely on relative ITS2 profile similarity. The larval infection experiment showed *A. kenti* larvae acquired both SCF049 and *C. proliferum* symbionts after 24 h and maintained them for the duration of the experiment (6 days), confirming both species can form a symbiosis with *Acropora* corals (Figure 5).

**FIGURE 5:**
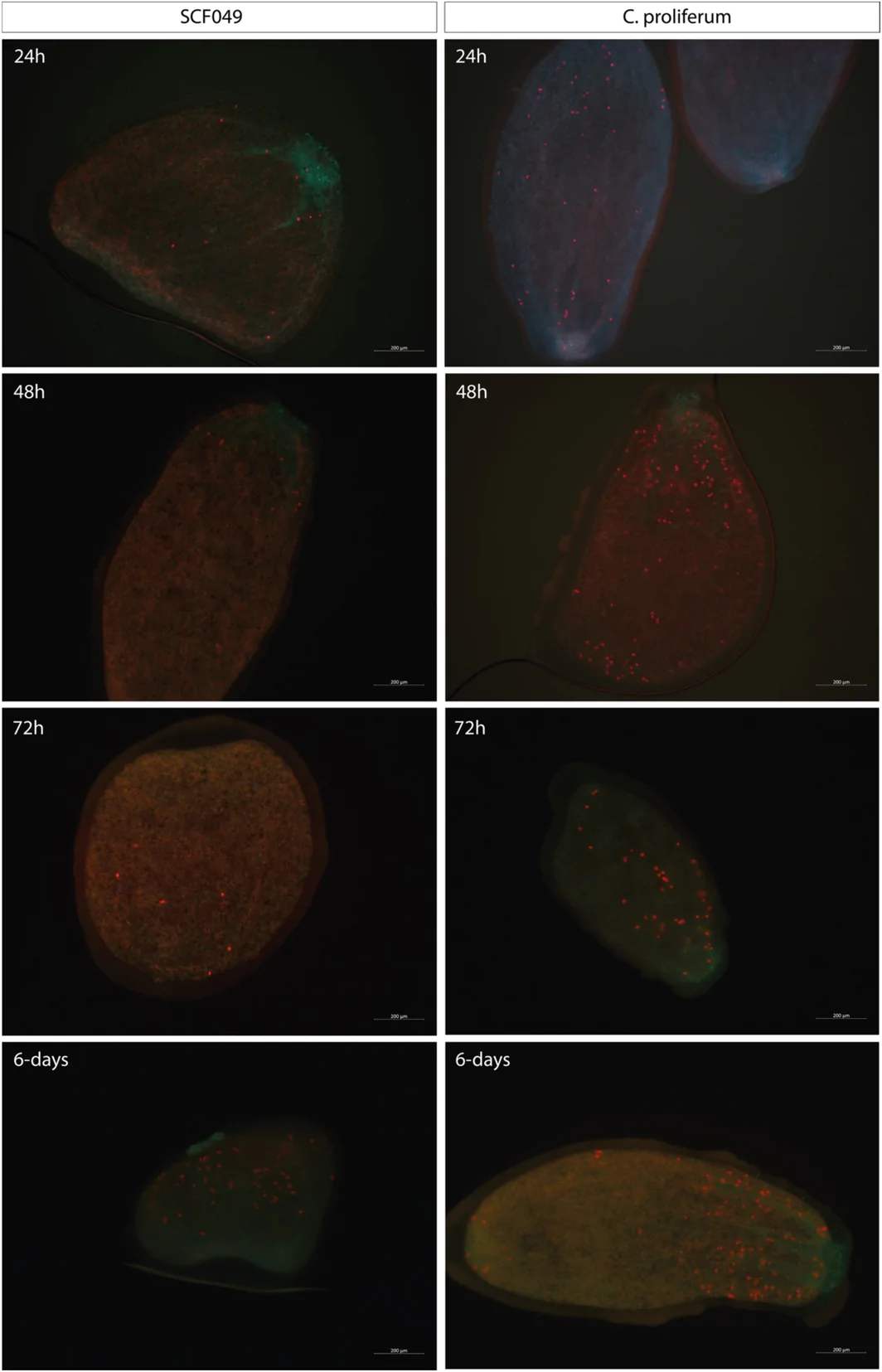
SCF049 and *C. proliferum* infection of *A. kenti* larvae. *A. kenti* larvae infected by SCF049 (left) and *C. proliferum* (right) at 24/48/72 h and 6 days post symbiont exposure. Red dots within the larval body are symbionts fluorescing under UV light.

A Principal Coordinate Analysis (PCoA) of GUniFraq distances illustrated the distribution of wild ITS2 profiles relative to the SCF049 and *C. proliferum* reference profiles (Figure 6). Several samples showed greater similarity to either reference profile, whereas a substantial proportion occupied unresolved positions relative to the two references. These assignments are intended as descriptive rather than taxonomic classifications, as ITS2 data alone cannot determine whether unresolved profiles represent divergent lineages, mixed symbiont assemblages, or other sources of intra‐ and inter‐genomic variation.

**FIGURE 6:**
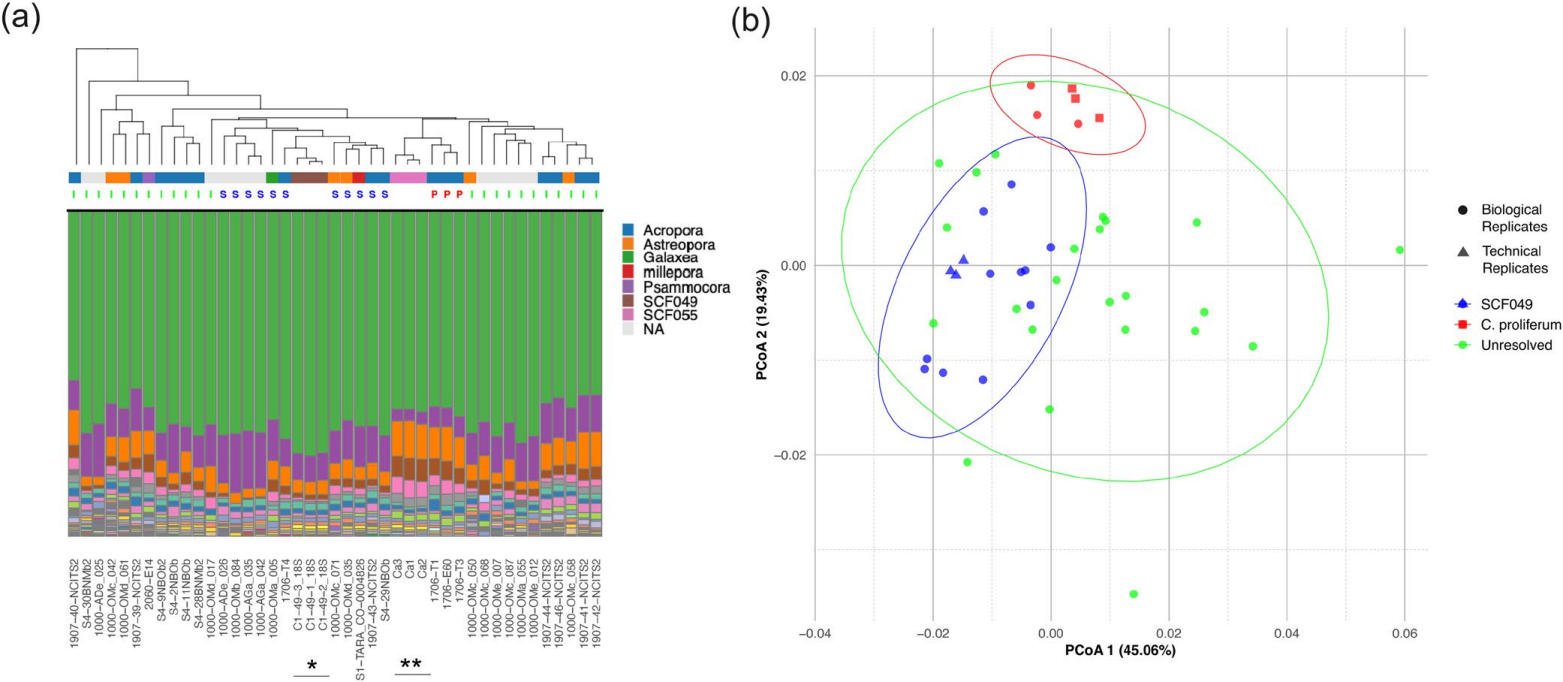
Genetic relatedness and community composition of SCF049 and *C. proliferum* ‐like Symbiodiniaceae based on SymPortal‐retrieved ITS2 profiles (a) Result of the Symportal search for SCF049 and *C. proliferum* like ITS2 profiles. UPGMA tree (k‐mer = 6 + GUniFraq (0.5) distances) in the top panel represents inter‐sample distances. The colored row indicates the genus of the host from which each sample was collected (according to Symportal metadata). Letters indicate the three categories (S = SCF049, P = *C. proliferum* , I = Intermediate) in which each sample has been assigned based on genetic distance from the ITS2 haplotype of the cultures of the two species, present in three technical replicates each (* = SCF049 culture/** = *C. proliferum* culture). Asterisks indicate only the culture haplotypes. The colored bars represent the relative abundances of ITS2 DIVs. (b) PCoA of GUniFrac (0.5) distances occurring among samples filtered during Symportal search for SCF049 and *C. proliferum* like ITS2 profiles.

### 3.4 SymPortal Screening—Geography

From the Symportal metadata information, the geographical ranges of the instances in which these DIV profiles have been previously found could be unveiled (Figure S4). Samples classified as SCF049‐like were collected in shallow water (1–4 m) of the tropical East and West Pacific and in the Persian Gulf. The three samples classified as *C. proliferum* were collected from the Great Barrier Reef (the type locality of the species). Intermediate profiles showed a geographical range largely overlapping that of SCF049‐like profiles. Within the limited dataset examined here, SCF049‐like profiles were retrieved more frequently than *C. proliferum* ‐like profiles, particularly from the Persian Gulf.

### 3.5 Formal Taxonomic Description

*Cladocopium proximale* Terzi and Nitschke sp. nov.

DESCRIPTION: Vegetative ovate cells with a mean length of 8.99 μm (±0.13 μm; CI) and a mean width of 8.03 μm (±0.13 μm; CI) (Figure 1a). Nucleotide sequences of the large ribosomal subunit rRNA gene, LSU (GeneBank PZ968583), intra‐genomic variants profile of the internal transcribed spacer 2 of the rRNA gene region, ITS2 (BioProject accession: PRJNA1535175), partial chloroplast large subunit, cp23S (GeneBank PZ968584), mitochondrial cytochrome b, cob (GeneBank PZ996727), mitochondrial cytochrome c oxidase subunit 1, cox1 (GeneBank PZ996728), and partial sequence of the non‐coding region of the Photosystem II protein D1 gene, psbA^ncr^ (GeneBank QB081653), genetically define this species.

HABITAT: Symbiont of scleractinian corals in the south‐west Pacific. Potentially the symbiont of scleractinian corals in the East Pacific and the Persian Gulf. Isolated from the tissue of *Pocillopora*, though it is not thought to be a common symbiont of this coral genus and may represent a chance culturing of a recently ingested or loosely associated cell.

HOLOTYPE: Aliquot of SCF049 preserved in 99% ethanol with the code BRI‐AQ1065207.

ETYMOLOGY: From the Latin “proximalis” which means near or close, highlighting its position as a closely related sister taxon to *Cladocopium proliferum*.

OTHER NOTES: Belonging to the C1 radiation, this species has the same dominant intra‐genomic ITS2 sequence “C1” as *Cladocopium goreaui*, *Cladocopium proliferum*, and *Cladocopium vulgare*. Contrary to *C. proliferum* , the culture SCF049 has higher “C1” sequence relative abundance (75% compared to 60%–65%). Moreover, *C. proximale* and *C. proliferum* differ consistently in the abundance of the second most abundant variant in their ITS2 intra‐genomic profile, C1c and C1b, respectively. Finally, sequence C1cb, the 7th most abundant sequence of *C. proliferum* , is absent in the *C. proximale* ITS2 profile. Also, some low abundant sequences are specific only to either one of the two profiles: C21, C3ew, C42aw, C1cv, and 281_C are only found in *C. proximale*, whereas C42a, C42ao, and C1cu are found only in the *C. proliferum* ITS2 profile. Live material of SCF049 is maintained in the Symbiont Culture Facility of the Australian Institute of Marine Science (Townsville, Australia).

## 4 Discussion

The Symbiodiniaceae are a diverse family consisting of at least 16 genus‐level lineages and an unknown number of species (Davies et al. 2023). Resolving their taxonomy is fundamentally important for understanding Symbiodiniaceae diversity, ecology, and coral–symbiont interactions (Nitschke et al. 2024). Here, we delimited *Cladocopium proximale* from its sister species *Cladocopium proliferum*.

### 4.1 Slowly‐Evolving Markers Fail to Delimit Closely Related Cladocopium Taxa

The complex genomic architecture of Symbiodiniaceae, including extensive intra‐genomic variation in multi‐copy markers such as ITS2, makes fine‐scale taxonomy challenging despite high overall genetic diversity (Davies et al. 2023). While an 85% similarity threshold in the D1/D2 region of the LSU sequence is standard for delimiting Symbiodiniaceae genera, no equivalent threshold exists for species beyond multi‐gene congruence (LaJeunesse et al. 2018), leaving species descriptions and the placement of closely related taxa uncertain (Davies et al. 2023). Symbiodiniaceae species are usually taxonomically described through a combination of slow‐ and fast‐evolving markers (LaJeunesse and Thornhill 2011). Even though some variation may be present in slow‐evolving regions among species of the same genus, the separation of closely related organisms, like *C. proximale* and *C. proliferum* , might be overlooked without considering regions of the genome with higher mutation rates.

### 4.2 The ITS2 and psbA^ncr^ Meta‐Haplotype Approach to Delimit Closely Related Species

To strengthen species delineation beyond using ITS2 alone and increase confidence in resolving closely related taxa, we employed an ITS2‐psbA^ncr^ meta‐haplotype approach (LaJeunesse and Thornhill 2011; Smith et al. 2017). Including a second hypervariable region is expected to increase the confidence with which two closely related species can be separated (LaJeunesse and Thornhill 2011). The psbA gene is present in chloroplasts on individual minicircles with multiple copies per chloroplast, while the ribosomal cistron occurs in tandem repeats in the nuclear genome (Moore et al. 2003). The non‐coding region of psbA is comprised of two hypervariable regions that flank four internal conserved cores (Moore et al. 2003). By sequencing a ~250 bp amplicon toward the 3′ end of the hypervariable region, we successfully generated a consensus sequence that serves as a reference psbA^ncr^ profile and consists of minicircle variants for each of *C. proximale* and *C. proliferum* pure cultures. Although the minicircle variants were shared by both species, the significant difference in their relative abundances enables the delineation of the cultures further than with ITS2 alone, and in combination, the meta‐haplotype data are diagnostic. While highly effective for cultures which contain low genetic diversity, applying such a meta‐haplotype approach to wild coral samples requires careful consideration due to the potential for multiple co‐occurring Symbiodiniaceae genotypes and thus inter‐ and intra‐genomic variants, which can obscure species‐level signals and complicate identification of dominant meta‐haplotypes (Smith et al. 2017).

Applying this approach to wild coral samples, we successfully identified the symbiont of *Galaxea* as *C. proximale*. However, *Psammocora* samples showed incongruent signal between markers. Their ITS2 profiles appeared intermediate between *C. proximale* and *C. proliferum* , consistent with either mixed symbiont communities or a cryptic lineage. In contrast, the psbA^ncr^ profiles were dominated by a distinct minicircle variant not present in either reference species, indicating these intermediate profiles likely represent a divergent evolutionary lineage. This highlights how multi‐organellar meta‐haplotype data can resolve ambiguous ITS2 profiles and provide greater confidence in taxa that are difficult to classify with ITS2 alone. Because psbA^ncr^ is plastid‐encoded and likely maternally inherited in Symbiodiniaceae, as in related microbial eukaryotes such as apicomplexans (Collier et al. 2025), it may further offer insight into lineage inheritance and connectivity, particularly as evidence for sexual reproduction in Symbiodiniaceae continues to accumulate (Chi et al. 2014; Figueroa et al. 2021).

### 4.3 Challenges During psbA^ncr^ Sequencing: Specificity, Inconsistency, and Artifacts

While earlier studies reported successful direct Sanger sequencing of full‐length psbA^ncr^ (Butler et al. 2023; LaJeunesse and Thornhill 2011), we were unable to replicate these results, even using the same culture, primers, and PCR conditions. Comparative analysis of the ~250 bp internal‐primer amplicons (metabarcoding and Sanger‐internal) versus the overlapping ~300 bp region obtained from full‐length Sanger psbA^ncr^ sequencing revealed discrepancies in the sequence variants recovered across methods. Notably, Sanger sequences previously published, and those generated in our study using primers targeting the entire ~1200 bp psbA^ncr^, clustered differently from those produced using internal primers, even when derived from the same DNA extract (i.e., SCF049 sequences generated from the same DNA voucher). This clustering discrepancy suggests the influence of PCR artifacts introduced during amplification with flanking primers. When denatured to single‐stranded DNA during PCR, the full‐length psbA^ncr^ exhibits highly complex secondary structure (Figure S1). In particular, C‐rich palindromic motifs can form strong hairpin structures (Aird et al. 2011; Viguera et al. 2001), especially during the annealing phase, increasing the likelihood of polymerase slippage and amplification error (Nelms and Labosky 2011; Singh et al. 2000). Because clean chromatograms are occasionally obtained for full‐length psbA^ncr^, these artifacts may arise during the earliest PCR cycles, become templates, and then be preferentially amplified (Butler et al. 2003; Polz and Cavanaugh 1998). As a result, reproducible but misleading sequence profiles may be produced, potentially masking genuine biological variation.

Altogether, a higher annealing temperature (60°C) in our PCR conditions, simpler secondary structure, and reduced amplicon length should minimize the likelihood of polymerase slippage (Aird et al. 2011; Butler et al. 2003). Although the behavior of secondary structures and their exact impact on PCR outcomes remains difficult to predict, these findings highlight their potential influence on sequencing results. The consistent discrepancies observed between full‐length and internal primer approaches underscore the importance of refining best practices when targeting structurally complex genomic regions. Ultimately, enhancing the accuracy of sequence variability assessments while minimizing the risk of interpreting artifacts as biologically meaningful variation requires further optimization in Symbiodiniaceae taxonomy. One promising direction is the application of real‐time sequencing enrichment approaches capable of spanning the full non‐coding region, which could recover true minicircle diversity, minimizing amplification bias in complex DNA samples (Burgess 2021).

Finally, the presence of suspected artifactual sequences in multiple sequence alignments can significantly distort phylogenetic inference (Acinas et al. 2005; Zhou et al. 2019). The primary diagnostic difference between *C. proximale* and *C. proliferum* in psbA^ncr^ is a single nucleotide polymorphism (SNP), which is completely obscured by the artifacts introduced in the full‐length psbA^ncr^ Sanger sequences. Environmental samples previously classified as *C. proliferum* are more likely *C. proximale*, based on the presence of this SNP. These findings underscore the critical importance of minimizing PCR artifacts and interpreting phylogenetic data with caution, particularly when working with closely related taxa and structurally complex genomic regions prone to amplification bias.

### 4.4 ITS2 Profile Search Provides Insights Into Biogeographical Distribution and Host Associations

ITS2 profiling is the most broadly utilized method for symbiont community description, especially in field applications (Davies et al. 2023; LaJeunesse and Thornhill 2011; Villela et al. 2024). It enables the high throughput characterization of the diversity and distribution of Symbiodiniaceae in relation to host population structure and environmental conditions (Marzonie et al. 2024). To retrieve ecological information, we identified samples presenting a similar ITS2 profile to *C. proximale* and *C. proliferum* . Nevertheless, based on GUniFrac distances, wild samples were predominantly intermediate of *C. proximale* or *C. proliferum* references. Whether these represent the coexistence of both species, an intermediate genotype due to interspecific hybridization, or a third distinct species is unclear without additional data such as psbA^ncr^. Notably, the intermediate samples exhibited broad dispersion in genetic ordination space, suggesting substantial cryptic diversity that has yet to be described. Future versions of Symportal or related pipelines and databasing would benefit from the standardization of multi‐locus assays.

The metadata associated with these samples retrieved from the Symportal database provides important ecological information. Considering the current application of *C. proliferum* in experimental evolution studies, and the potential utilization of *C. proximale*, obtaining ecological information is imperative for planning future interventions. In fact, knowing geographical and depth ranges (Davies et al. 2023), as well as possible host‐symbiont pairs, will contribute to correctly directing efforts, increasing intervention success and reducing time and costs (Davies et al. 2023; Nitschke et al. 2024). *C. proximale*‐like profiles were retrieved from a range of Scleractinia and Hydrozoa, whereas fewer samples could be classified as *C. proliferum* ‐like, limiting the ecological information available for this lineage. Although *C. proliferum* ‐like profiles were retrieved only from *Acropora* corals in the datasets examined here, *C. proliferum* is likely to share the host‐generalist characteristics reported for other *Cladocopium* species in previous studies (Butler et al. 2023, 2003; Scharfenstein et al. 2022).

The larval infection experiment showed both *Cladocopium* species successfully infected *Acropora kenti* larvae and were retained throughout the 6‐day experiment, indicating both lineages can establish early symbioses with the same coral host. However, symbiont uptake and persistence during this early larval stage should not be interpreted as evidence of long‐term compatibility with adult corals, as host–symbiont associations may become increasingly selective during development through the winnowing of initially acquired symbiont diversity (Chan et al. 2019). While qualitative, these findings demonstrate comparable capacity for initial host colonization and short‐term persistence, although longer‐term experiments would be required to determine compatibility at later developmental or adult stages.

Finally, it is important to highlight the presence of *C. proximale‐*like ITS2 profiles in the Persian Gulf, which is beyond the distribution reported for *C. proliferum* (Butler et al. 2023). Corals and symbionts of the Persian Gulf are known to be adapted to higher temperature conditions (D'Angelo et al. 2015), thus *C. proximale* might represent an important source of functional novelty within assisted evolution frameworks, as applied to the thermally resilient strain (SCF055) of *C. proliferum* .

### 4.5 Taxonomic Resolution as a Foundation for Applied Symbiont Research

The separation of *C. proximale* and *C. proliferum* into distinct taxa is important in the context of coral reef restoration (Davies et al. 2023) as different species may respond differently to experimental evolution targeting thermal tolerance (Chakravarti and van Oppen 2018). Because their geographical and ecological ranges overlap, these sister species may also provide a valuable system for experimental studies investigating interactions between closely related symbionts, including symbiont competition. Importantly, these two species and their type cultures are potentially the two most closely related cultures in existence. They were isolated from different hosts, at different times, and at different locations. For these reasons, they provide a unique opportunity to study Symbiodiniaceae speciation processes, and the potential role of sexual reproduction and ongoing hybridization in creating genetic diversity in experimental evolution efforts that aim to enhance their thermal tolerance. Any application of these cultures in restoration would, however, require further assessment of their relevant traits, host compatibility, and potential ecological risks. At present, their value lies primarily as a well‐characterized experimental system for future research. Considering these implications, along with supporting morphological and genetic data and the value of this information during restoration applications, we propose a separation into two different, formally described species.

## Author Contributions

**Giorgio Terzi:** data curation (lead), formal analysis (lead), investigation (equal), methodology (equal), software (equal), writing – original draft (lead). **Matthew R. Nitschke:** conceptualization (lead), funding acquisition (lead), investigation (equal), methodology (lead), project administration (lead), software (equal), supervision (lead), validation (lead), writing – review and editing (equal). **Madeleine J. H. van Oppen:** conceptualization (equal), funding acquisition (lead), investigation (equal), project administration (equal), supervision (equal), writing – review and editing (equal). **Davide Seveso:** supervision (equal), validation (equal), writing – review and editing (equal).

## Funding

The authors have nothing to report.

## Conflicts of Interest

The authors declare no conflicts of interest.

## Supporting information


**Figure S1:** Full length psbA non‐coding region multiple sequence alignment. Highlighted the ~350 bp this region used to generate phylogenetic inference.

**Figure S2:** Predicted secondary structure and associated free energy for *C. proliferum* (culture SCF055) full‐length psbA^ncr^ and ASV1 sequences.

**Figure S3:** Predicted secondary structure and associated free energy for SCF049 LSU, cob, cox1 and cp23s sequences.

**Figure S4:** World map showing in the locations from which the Symportal retrieved samples have been collected. Piecharts indicate the percentage of sequences assigned to the three categories C. *proximale*, *C. proliferum* and mixed community in each study based on genetic distance from the ITS2 haplotype of the two species. A zoom‐in has been done on the Persian Gulf region for better separation of single instances.

**Table S1:** Accession numbers to Genebank nucleotide database for slow‐evolving markers sequences of the eleven members of Cladocopium and Halluxium sp. used to build the low‐resolution tree (Figure 1c).

**Table S2:** Accession numbers to Genebank nucleotide database for 21 non‐coding regions of psbA sequences (generated with flanking primers) of Cladocopium used to build the high‐resolution tree in Figure 4a.

**Table S3:** Metadata for *Galaxea* and *Psammocora* samples.

## Data Availability

The data that support the findings of this study are openly available in Cladocopium‐proximale‐data at https://github.com/Gioterzi/‐Cladocopium‐proximale‐data.
