# Supplementary material for: Formal Description of Cladocopium proximale sp. nov., a Coral Photosymbiont From the C1 Radiation
Source: Ecol Evol. 2026 Nov 5;16(11):e74569. doi: 10.1002/ece3.74569 (PMC13739419; doi:10.1002/ece3.74569)
Supplement: Supplementary file 1 [file ECE3-16-e74569-s001.docx]

| Sample | Genera | Species | Collection date | Collection site | Lat/Lon | Permit | Preservation |
| --- | --- | --- | --- | --- | --- | --- | --- |
| *Gal-509* | Galaxea | cf. acrhelia | 2024-02-23 00:00:00 | Falcon Reef | 18.763750, 146.532269 | G22/46479.1 | LN2 |
| *Psa-36* | Psammocora | columna | 2022-05-25 00:00:00 | Fantome Reef | 18.6649948, 146.5053023 | G21/38062.1 | LN2 |
| *Psa-44* | Psammocora | columna | 2022-05-25 00:00:00 | Fantome Reef | 18.6649948, 146.5053023 | G21/38062.1 | LN2 |
| *Psa-109* | Psammocora | columna | 2022-05-25 00:00:00 | Fantome Reef | 18.6649948, 146.5053023 | G21/38062.1 | LN2 |
| *Psa-113* | Psammocora | columna | 2022-05-25 00:00:00 | Fantome Reef | 18.6649948, 146.5053023 | G21/38062.1 | LN2 |

Table S3. Metadata for Galaxea and Psammocora samples
