# Supplementary material for: Formal Description of Cladocopium proximale sp. nov., a Coral Photosymbiont From the C1 Radiation
Source: Ecol Evol. 2026 Nov 5;16(11):e74569. doi: 10.1002/ece3.74569 (PMC13739419; doi:10.1002/ece3.74569)
Supplement: Supplementary file 2 [file ECE3-16-e74569-s003.docx]

| **Genus** | **Species** | **LSU** | **cp23S** | **Cox1** | **Cob** |
| --- | --- | --- | --- | --- | --- |
| *Halluxium* | *sp.* | MT015652 | MT022024 | JN557895 | MT021980 |
| *Cladocopium* | *thermophilum* | OM836135 | KP234523 | KR996472 | KR996513 |
| *Cladocopium* | *pacificum* | MW711733 | MW711736 | MW713057 | MW713375 |
| *Cladocopium* | *infistulum* | MT755914 | MT755915 | MT764187 | MT764191 |
| *Cladocopium* | *latusorum* | MW711731 | MW711735 | MW713056 | MW713374 |
| *Cladocopium* | *goreaui* | FJ529523 | FJ529545 | MT764188 | KF206028 |
| *Cladocopium* | *madreporum* | OQ361828 | OQ361801 | OQ359941 | OQ260007 |
| *Cladocopium* | *patulum* | OQ361830 | OQ361803 | OQ359943 | OQ260009 |
| *Cladocopium* | *sodalum* | OQ361832 | OQ361804 | OQ359944 | OQ260010 |
| *Cladocopium* | *vulgare* | OQ361829 | OQ361802 | OQ359942 | OQ260008 |
| *Cladocopium* | *proliferum (SCF055)* | This study | This study | This study | This study |
| *Cladocopium* | *proximale (SCF049)* | This study | This study | This study | This study |

Table S1. Accession numbers to Genebank nucleotide database for slow-evolving markers sequences of the eleven members of Cladocopium and Halluxium sp. used to build the low-resolution tree (Figure 1c).
