# Supplementary material for: Formal Description of Cladocopium proximale sp. nov., a Coral Photosymbiont From the C1 Radiation
Source: Ecol Evol. 2026 Nov 5;16(11):e74569. doi: 10.1002/ece3.74569 (PMC13739419; doi:10.1002/ece3.74569)
Supplement: Supplementary file 4 [file ECE3-16-e74569-s006.docx]

| Species | Sample | psbAncr A.N. |
| --- | --- | --- |
| *vulgare* | A03_392 | OQ359934 |
| *vulgare* | A03_311 | OQ359933 |
| *vulgare* | Pho09_239 | OQ359932 |
| *vulgare* | A03_395 | OQ359931 |
| *vulgare* | Palau | OQ359930 |
| *vulgare* | NewCal | OQ359928 |
| *proliferum* | JPB08 | OQ359935 |
| *proliferum* | 55 | OQ359937 |
| *proliferum* | A03 | OQ359938 |
| *proliferum* | A02 | OQ359939 |
| *proliferum* | Zam | OQ359940 |
| *latusorum* | Haw09_014 | MW819779 |
| *latusorum* | Haw09_012 | MW819778 |
| *latusorum* | SML07_60 | MW819777 |
| *latusorum* | TW_117 | MW819773 |
| *pacificum* | VR19_LJ05 | OR887589 |
| *pacificum* | VR19_LJ13 | OR887591 |
| *pacificum* | VR19_LJ20 | OR887593 |
| *pacificum* | VR19_LJ31 | OR887595 |
| *proliferum* | SCF055 | This study |
| SCF049 | SCF049 | This study |

Table S2. Accession numbers to Genebank nucleotide database for 21 non-coding regions of psbA sequences (generated with flanking primers) of Cladocopium used to build the high-resolution tree in Figure 4a.
